# Supplementary figures and images for: Genome-Wide Identification and Expression of the Paulownia fortunei MADS-Box Gene Family in Response to Phytoplasma Infection
Source: Genes (Basel). 2023 Mar 11;14(3):696. doi: 10.3390/genes14030696 (PMC10048600; doi:10.3390/genes14030696)

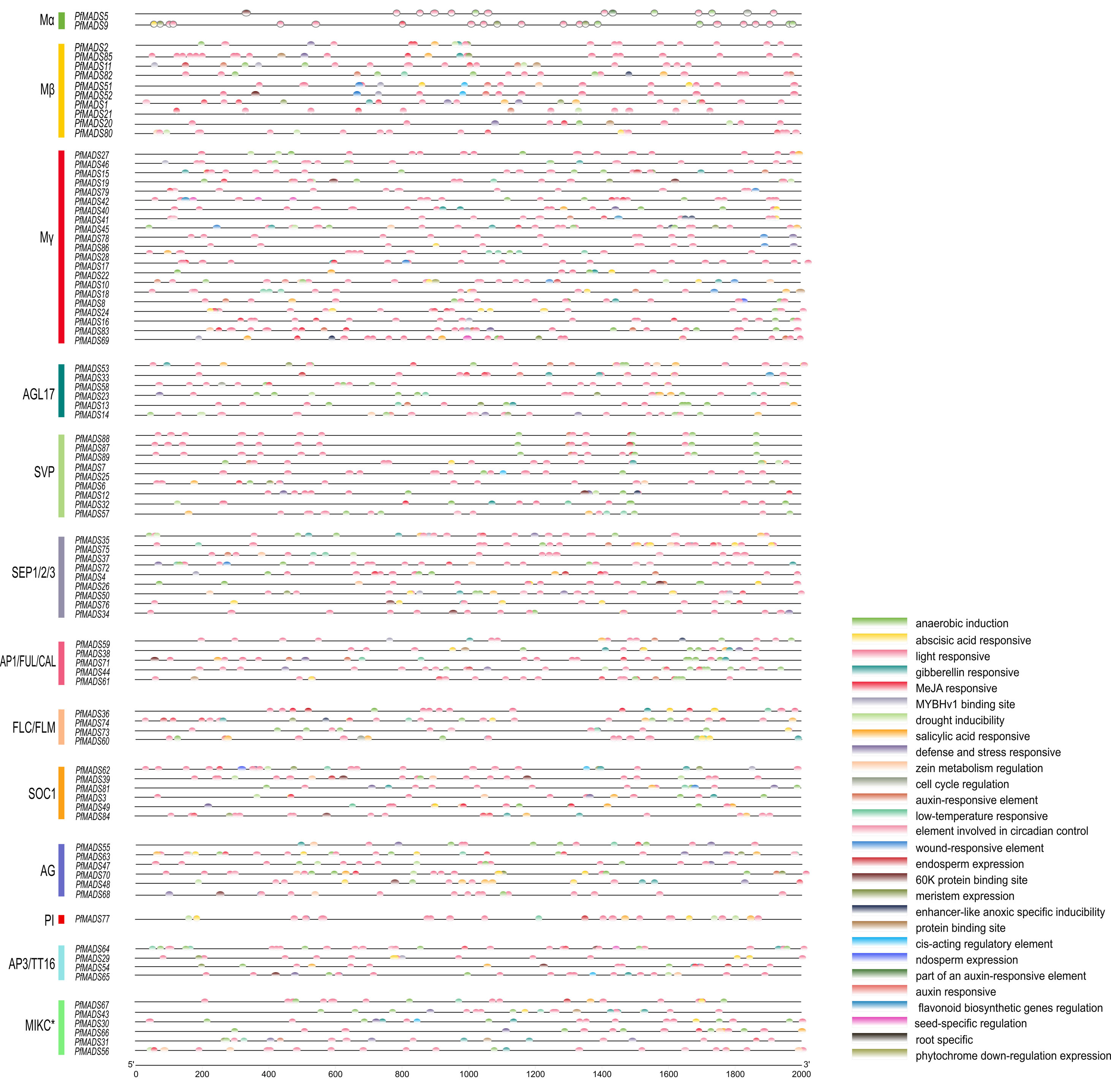

Supplement: Supplementary file 1 [file genes-14-00696-s001.zip › Fig S2.jpg]

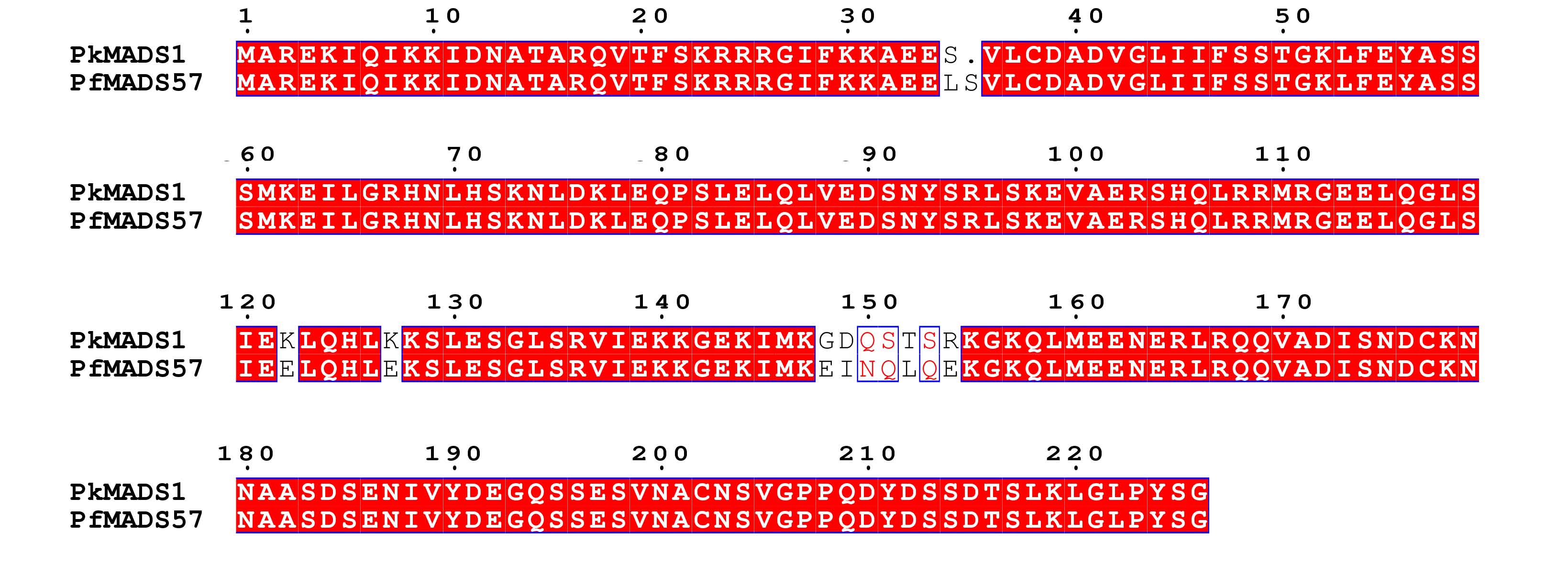

Supplement: Supplementary file 1 [file genes-14-00696-s001.zip › Fig S3.jpg]

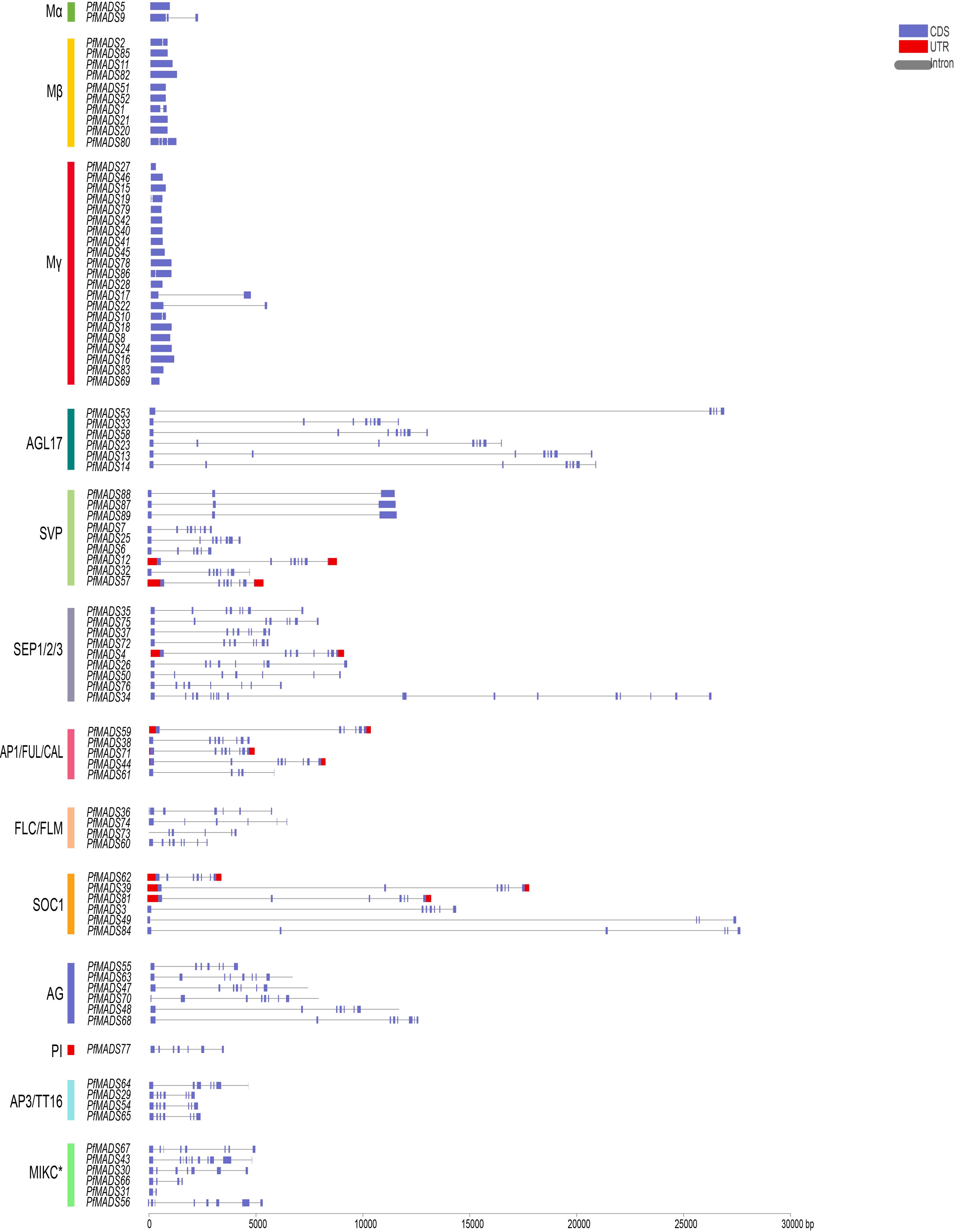

Supplement: Supplementary file 1 [file genes-14-00696-s001.zip › Fig S1.jpg]
